# *Coxiella* and *Bartonella* spp. in bats (Chiroptera) captured in the Brazilian Atlantic Forest biome

**DOI:** 10.1186/s12917-018-1603-0

**Published:** 2018-09-10

**Authors:** Michelle Santos Ferreira, Alexandro Guterres, Tatiana Rozental, Roberto Leonan Morim Novaes, Emmanuel Messias Vilar, Renata Carvalho de Oliveira, Jorlan Fernandes, Danielle Forneas, Adonai Alvino Junior, Martha Lima Brandão, José Luis Passos Cordeiro, Martín Roberto Del Valle Alvarez, Sergio Luiz Althoff, Ricardo Moratelli, Pedro Cordeiro-Estrela, Rui Cerqueira da Silva, Elba Regina Sampaio de Lemos

**Affiliations:** 1 Laboratório de Hantaviroses e Rickettsioses, Pavilhão Helio e Peggy Pereira, 1 Pavimento, Instituto Oswaldo Cruz, Fundação Oswaldo Cruz, Avenida Brasil 4365, Manguinhos, Rio de Janeiro, RJ Brazil; 2 Universidade Federal do Rio de Janeiro, Av. Pedro Calmon, 550, Cidade Universitária, Rio de Janeiro, Rio de Janeiro, RJ Brazil; 3 Laboratório de Mamíferos, Departamento de Sistemática e Ecologia, Centro de Ciências Exatas e da Natureza, Universidade Federal da Paraíba, Campus I, Castelo Branco, João Pessoa, PB Brazil; 4 Fundação Oswaldo Cruz, Fiocruz Mata Atlântica, Estrada Rodrigues Caldas, 3400, Taquara, Rio de Janeiro, RJ Brazil; 5 Departamento de Ciências Biológicas, Universidade Estadual de Santa Cruz, Rodovia Ilhéus - Itabuna, Km. 16 Salobrinho, Ilheus, BA Brazil; 6 Departamento de Ciências Naturais, Laboratório de Biologia Animal, Fundação Universidade Regional de Blumenau, Ccen, Dcn. FURB - Fundação Universidade Regional de Blumenau Itoupava Seca, Blumenau, SC Brazil; 7 Laboratório de Vertebrados, Departamento de Ecologia, Instituto de Biologia, Universidade Federal do Rio de Janeiro, Av. Pedro Calmon, 550, Cidade Universitária, Rio de Janeiro, RJ Brazil

**Keywords:** *Coxiella burnetii*, *Bartonella*, Zoonotic bacterial agent, Mammals, Atlantic Forest hotspot, Brazil

## Abstract

**Background:**

The role of bats as reservoirs of zoonotic agents, especially pathogenic bacteria such as Bartonella and Coxiella, has been discussed around the world. Recent studies have identified bats as potential hosts of species from the proteobacteria phylum. In Brazil, however, the role of bats in the natural cycle of these agents is poorly investigated and generally neglected. In order to analyze the participation of bats in the epidemiology of diseases caused by *Bartonella*, *Coxiella*, *Rickettsia*, *Anaplasma* and *Ehrlichia*, we conducted a descriptive epidemiological study in three biogeographic regions of the Brazilian Atlantic Forest.

**Results:**

Tissues of 119 bats captured in preserved areas in the states of Rio de Janeiro, Bahia and Santa Catarina from 2014 to 2015 were submitted to molecular analysis using specific primers. *Bartonella* spp. was detected in 22 spleen samples (18.5%, 95% CI: 11.9–26.6), whose phylogenetic analysis revealed the generation of at least two independent clusters, suggesting that these may be new unique genotypes of *Bartonella* species. In addition, four samples (3.4%, 95% CI: 0.9–8.3) were positive for the *htpAB* gene of *C. burnetii* [spleen (2), liver (1) and heart (1)]. *Rickettsia* spp., *Anaplasma* and *Ehrlichia* were not identified. This is the first study reporting *C. burnetii* and *Bartonella* spp. infections in bats from the Atlantic Forest biome.

**Conclusions:**

These findings shed light on potential host range for these bacteria, which are characterized as important zoonotic pathogens.

**Electronic supplementary material:**

The online version of this article (10.1186/s12917-018-1603-0) contains supplementary material, which is available to authorized users.

## Background

Bacteria transmitted by arthropods belonging to the genera *Rickettsia*, *Bartonella*, *Coxiella*, *Ehrlichia* and *Anaplasma* are pathogens of domestic and wild animals as well as humans. These agents cause diseases that may be severe and have a widespread geographic distribution, such as bartonelosis, ehrlichiosis, anaplasmosis, spotted fever, and Coxielosis/Q fever [1–4]. *Bartonella* spp. (proteobacteria α2 group), an intracellular hemotropic bacterium that grows fastidiously, is transmitted mainly by flea bites [4]. *Coxiella burnetii* (proteobacteria γ group), the causative agent of Q fever/Coxiellosis, is a highly infectious zoonotic intracellular bacterium transmitted by inhalation of aerosols or contaminated excreta materials. Ticks are suspected of having a role in the transmission of this pathogen among animals [5]. *Rickettsia* (proteobacteria α1 group) is a representative genus group of pathogenic or non-pathogenic intracellular bacteria transmitted by ticks, mites, lice and fleas [2]. *Ehrlichia* and *Anaplasma* (proteobacteria α1 group), which are known to cause diseases in animals and humans, are kept in the wild in a cycle involving mammals and arthropods [6]. In recent years, studies have pointed to bats as hosts of these proteobacteria around the world [7–11]. Their increasing diversity and apparent clade-specific association for *Bartonella* spp. [12] encourages increasing inventory and surveillance efforts, especially in sylvatic environments to better understand their natural transmission cycles.

Bats (order Chiroptera) occur in all continents except Antarctica [13]. Among mammals, they are outnumbered only by rodents in species richness but surpass all other groups in dietary diversity, including fruit eaters, nectar feeders, insectivores, carnivores, blood feeders and omnivores. In biodiversity hotspots such as Brazil’s Atlantic Forest, bats are the most diverse and abundant mammals, and they represent wild vertebrates that interact with humans [14] especially in urban areas [15]. The role of bats as important hosts for emerging human diseases has gained the attention of the scientific community. They are recognized for harboring viral infectious agents and, less recognizably, bacterial agents of public health importance [16–18].

In Brazil, the vespertilionid bat species *Histiotus velatus* (tropical big-eared brown bat), and the phyllostomids *Carollia perspicillata* (Seba’s short-tailed bat) and *Desmodus rotundus* (common vampire bat) were considered reservoirs of rickettsiae in an experimental study in the 1950s [19]. After more than 50 years, in the city of São Paulo, molossid, vespertilionid and phyllostomid bats were seroreactive to, at least, one rickettsial antigen of the spotted fever group [20]. In Queensland, Australia, in 2014, the DNA of *C. burnetii* was found in bat urine pools of a *Pteropus* (Pteropodidae) [8]. This finding might be indicative of the potential role of these animals as a source of infection for humans and other animal species through the inhalation of contaminated aerosols. Probably related to their sporulation process, *C. burnetii* survives for long periods in the environment, and inhalation is characterized as the main mechanism by which this microorganism is transmitted [21–23].

In addition, there are studies around the world in which from different species of *Bartonella* has been detected in bats for example, in, Peru [24], Kenya [25], United Kingdom [26], Guatemala [27], Nigeria [7], Puerto Rico [28], Vietnam [9], Costa Rica [10] and Taiwan [29]. More recently, strains of *Bartonella mayotimonensis*, a recognized human pathogen, were identified and isolated from bats in the northern hemisphere [30]. In Brazil, up to now, there is one study associating *Bartonella* with bats [11]. However, the real role of bats as hosts and maintainers of the natural cycle of these bacteria in nature remains unknown. Studies proving associations between bats and *C. burnetii*, *Ehrlichia* and *Anaplasma* have not yet been reported.

Considering the growing importance of bats as potential reservoirs and transmitters of different pathogens around the world as well as the paucity of investigations about the role of bats in the dissemination of proteobacteria pathogens, the present study provides information about the circulation of these zoonotic bacteria in bats captured in three Atlantic Forest localities in Brazil where notifications of Brazilian spotted fever, Q fever and bartonelosis have been reported.

## Methods

### Study areas and sample collection

**Fig. 1:**
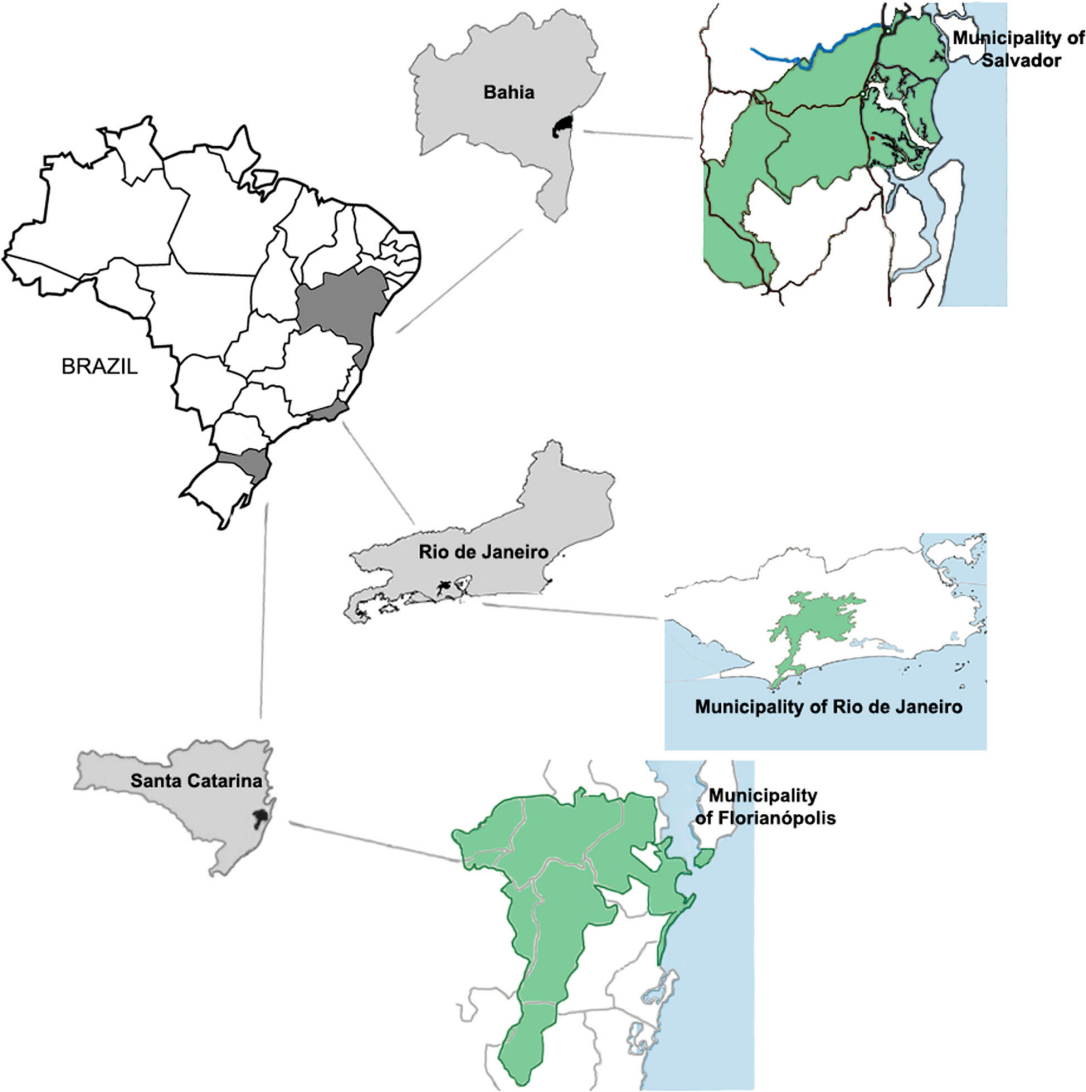
Geographic location of sampling sites in the Atlantic Forest of Brazil

The study was conducted in three Brazil’s Atlantic Forest localities: Oswaldo Cruz Foundation (Fiocruz) Atlantic Forest Biological Station (EFMA; 22°56′22.9"S 43°24′12.2"W), Pedra Branca Massif, Jacarepaguá, which is a metropolitan area of the city of Rio de Janeiro (RJ); city of Igrapiúna, southern region of Bahia (BA), which is within the Environmental Protection Area (APA) of Pratigi (13°50′43.3"S 39°16′17.0"W); Serra do Tabuleiro State Park (PEST; 27°44′30.8"S 48°48′26.7"W), which is located in the central-eastern region of Santa Catarina state (SC) in the metropolitan area of the city Florianópolis (Fig. 1). The vegetation of the three sampling areas are composed by lowland humid forest areas from three different biogeographic regions of the Atlantic Forest biome.

From December 2013 to May 2015, expeditions were carried out and bats were captured using 10 ground-level mist nets (9 × 3 m) each night in forest edges or along pre-existing trails. Permits for field collection were granted by an Brazilian Institute of Environment and Renewable Natural Resources (IBAMA) license under process numbers 19,037–1; Santa Catarina’s Environment Foundation (FATMA) no. 043/2014/GERUC/DPEC, Chico Mendes Biodiversity Conservation Institute (ICMBio/SISBIO) no. 26934–1 and no. 17131–4 (ICMBio/SISBIO). Adult males and non-pregnant and non-lactating adult females were euthanized. The euthanasia method consisted of cardiac puncture exsanguination performed after the anesthesia procedure of the animal, according to previously established protocols [31]. Efforts were made to minimize animal suffering following protocols approved by the Institutional Ethics Committee on Animal Research (CEUA) of the Oswaldo Cruz Foundation under process numbers CEUA P.62/11–3 (LW-68/12) and P.42/12–1 (LW-81/12).

The sex, age class and biometry of each bat were registered. Bats tissues (i.e., kidney, liver, spleen, lung and heart) were sampled and preserved in absolute ethanol. Tissue samples were obtained in accordance with recommended safety procedures and followed previously established standard protocols [32]. Bat species was identified following the identification keys available in Gardner [33] and nomenclature in Nogueira et al. [34]. Voucher specimens were deposited at scientific collections from each region where the study was performed: Federal University of Rio de Janeiro, for bats collected in Rio de Janeiro; State University of Santa Cruz, for bats from Bahia; Foundation University of Blumenal and in the Collection of Mammals of the Federal University of Paraíba, for bats collected in Santa Catarina state. Prevalences and approximated confidence intervals (CIs) were calculated using the package “binom”. To evaluate the influence of the sex-ratio on the positivity we use a Fisher exact’s test.

### Nucleic acid extraction

The DNA extraction procedures were performed in laminar flow biosafety cabinet in a Biosafety Level 3 laboratory (Vecobiosafe, Veco, Campinas, SP, Brazil). DNA was extracted from 10 mg of each bat tissue using the commercial QIAamp DNA Mini Kit (QIAGEN, Valencia, CA, USA) according to the manufacturer’s instructions. The final volume of 100-μl obtained after elution in AE buffer (QIAGEN, Valencia, CA, USA). Negative controls using nuclease-free water were included in each extraction to check for DNA contamination.

Spleen tissues were investigated for all agents covered in this study; however, in an attempt to identify complementarity of information for *Coxiella burnetii* research, other tissues were tested as well.

### PCR amplification

Conventional polymerase chain reaction (PCR) assays was used to detect the target genes. The gene for which each agent investigated in this study was tested is listed below and demonstrated in Table 1. *Bartonella* spp., *gltA* gene [35]. *Coxiella burnetii,* bacterial-specific primers designed to amplify the IS1111 gene [36, 37]. *Rickettsia* spp., a partial sequence of the *gltA* gene [35]. *Ehrlichia* and *Anaplasma* bacterial,16S rRNA gene [38].

**Table 1:** Oligonucleotide primers used for screening bat samples of *Coxiella burnetii*, *Bartonella* spp., *Rickettsia* spp., *Ehrlichia* spp. and *Anaplasma* spp.

Pathogen	Target gene	Oligonucleotide primer	Primer sequence (5′ – 3′)	Amplicon size (bp)	Cycling conditions	Reference
*Coxiella burnetii*	IS1111	Outer primer F Outer primer R Nested primer F Nested primer R	TATGTATCCACCGTAGCCAGC CCCAACAACACCTCCTTATTC AAGCGTGTGGAGGAGCGAACC CTCGTAATCACCAATCGCTTCGTC	687 bp 440 bp	95 °C for 5 min, 40 cycles of 95 °C for 30s, 60 °C for 30s, 72 °C for 1 min, final extension of 72 °C for 7 min 95 °C for 5 min, 30 cycles of 95 °C for 30 s, 66 °C for 30 s, 72 °C for 30 s, final extension of 72 °C for 5 min	[36] [35, 37]
*Bartonella* spp.	gltA	Outer primer F Outer primer R	GCTATGTCTGCVTTCTATCAYGA AGAACAGTAAACATTTCN GTHGG	731 bp	95 °C for 10 min, 35 cycles of 95 °C for 30s, 58 °C for 30s, 72 °C for 1 min, final extension of 72 °C for 8 min	[35]
*Rickettsia* spp.	gltA	Outer primer F Outer primer R Nested primer F Nested primer R	CATCCTATGGCTATTATGCTTGC TATACTCTCTATG(T/A)AC(A/G)T(A/G)ACC CTTACCGCTATTAGAATGATTGC GAGCGA(T/G)AGCTTCAAG(T/C)TCTAT	885 bp 572 bp	95 °C for 10 min, 30 cycles of 95 °C for 30 s, 55 °C for 40 s, 72 °C for 55 s, final extension of 72 °C for 10 min 95 °C for 7 min, 25 cycles of 95 °C for 30 s, 63 °C for 30 s, 72 °C for 35 s, final extension of 72 °C for 10 min	[35] [35]
*Ehrlichia s*pp. /*Anaplasma* spp.	16 s rRNA	Outer primer F Outer primer R	GGTACCYACAGAAGAAGTCC TGCACTCATCGTTTACAG	345 bp	95 °C for 3 min, 35 cycles of 95 °C for 15 s, 55 °C for 30 s, 72 °C for 30s, final extension of 72 °C for 5 min	[38]

The mixture to each reaction contained 2.5 μl of 10X PCR buffer, 0.6 μl of 10 mM of each primer, 0.75–2 μl of 50 mM MgCl2, 0.25 μl deoxynucleotides (20 mM of each deoxynucleotide triphosphate), 0.1 μl Taq Platinum DNA polymerase (5 U/μl Invitrogen, Carlsbad, CA, USA) and nuclease-free water (Promega, Madison, WI, USA) to obtain a final volume of 25 μl. The volumes of the DNA sample varied as a function of the primer used. To *Bartonella* spp. was used 3 μl; to *Rickettsia* spp., 3 μl for PCR 1 and 2 μl for nested PCR; to *C. burnetii,*4 μl for PCR 1 and 2 μl for nested PCR; to *Ehrlichia* and *Anaplasma* spp., 2.5 μl. Volumes pre-established in a previous study [35]. Negative controls using nuclease-free water were included in each PCR assay to check for possible DNA contamination. Genomic DNA extracted from positive clinical samples from the National Reference Laboratory for Rickettsioses were used as positive controls. PCR amplification was subjected to a 1.5% agarose gel, stained with GelRed™ (Biotium, Hayward, CA, USA).

### DNA sequencing and phylogenetic analyses

Appropriately sized fragments were purified using Illustra GFX PCR DNA and Gel Band Purification® kit (GE Healthcare, Buckinghamshire, UK), direct nucleotide sequencing amplicon was performed using the BigDye Terminator v3.1 Cycle Sequencing kit, and purification was performed using the BigDye® X-Terminator Purification kit (Applied Biosystems, Foster City, CA, USA), according to the manufacturer’s recommendations. The analyses of the amplicons were performed in an ABI Prism 3730XL with 96 capillaries (Applied Biosystems) and the nucleotide sequences were analyzed using MEGA7 software (downloaded from www.megasoftware.net). A consensus sequence for each bacterial genome was derived from contiguous sequences assembled with the same software.

Multiple sequence alignments were done with sequences obtained from this study and sequences from the GenBank using MUSCLE in the SeaView v.4 program [39]. The best-fit evolutionary model was determined using MEGA version 7 by the Bayesian Information Criterion [40]**.** The phylogenetic tree was estimated using two methods: (a) Maximum Likelihood using PhyML implemented in SeaView v.4 [41], where the statistical support of the clades was measured by a heuristic search with 1000 bootstrap replicates; and (b) a Bayesian Markov Chain Monte Carlo (MCMC) method implemented in MrBayes v.3.2.6 [42]. The Bayesian analysis consisted of two simultaneous independent runs of 10 million MCMC generations (burn-in of 25%).

## Results

### Bat sampling

A total of 119 adult bats belonging to 21 species were sampled; *n* = 44 from EFMA/RJ; *n* = 47 from APA Pratigi/BA and *n* = 28 from PEST/SC (Table 2). The species sampled and their abundances are as follows: *Carollia perspicillata* (*n* = 34), *Desmodus rotundus* (15), *Artibeus lituratus* (14), *Sturnira lilium* (12), *Artibeus fimbriatus* (7), *Rhinophylla pumilio* (7) *Artibeus planirostris* (5), *Dermanura cinerea* (4), *Phyllostomus discolor* (4), *Artibeus obscurus* (2), *Glossophaga soricina* (2), *Myotis nigricans* (2), *Sturnira tildae* (2), *Vampyressa pusilla* (2), *Anoura caudifer* (1), *Chiroderma doriae* (1), *Lonchophylla peracchii* (1), *Micronycteris minuta* (1), *Micronycteris* sp. (1), *Phyllostonus hastatus* (1), and *Trinycteris nicefori* (1).

**Table 2:** Number of bats collected per locality (n), total number of bats (N), and infected bats (p), 95% confidence intervals of prevalences (CI) by *Bartonella* spp. and *Coxiella burnetii*

Family: Sub-family	Localities				PCR assay		
	Jacarepagua/RJ n/N(%)	APA Pratigi/BA n/N(%)	PEST/SC n/N(%)	Total bats N(%)	*Bartonella* positive p/N(%;CI)	*Coxiella* positive p/N(%; CI)	*Bartonella* and *Coxiella* positive p/N(%;CI)
Phyllostomidae: Carolliinae							
*Carollia perspicillata*	4/34(11.8)	23/34(67.7)	7/34(20.6)	34(28.6)	5/34(14.7; 4.9–31.0)	NS	NA
*Rhinophylla pumilio*	0/7(0)	7/7(100)	0/7(0)	7(5.9)	1/7(14.3; 0.3–57.8)	NS	NA
Phyllostomidae: Desmodontinae							
*Desmodus rotundus*	15/15(100)	0/15(0)	0/15(0)	15(12.6)	6/15(40; 16.3–67.7)	NS	NA
Phyllostomidae: Glossophaginae							
*Anoura caudifer*	0/1(0)	0/1(0)	1/1 (100)	1(0.8)	NS	NS	NA
*Glossophaga soricina*	2/2(100)	0/2(0)	0/2(0)	2(1.7)	NS	NS	NA
*Lonchophylla peracchii*	1/1(100)	0/1(0)	0/1(0)	1(0.8)	NS	NS	NA
Phyllostomidae: Glyphonycterinae							
*Trinycteris nicefori*	0/1(0)	1/1(100)	0/1(0)	1(0.8)	NS	NS	NA
Phyllostomidae: Micronycterinae							
*Micronycteris minuta*	1/1(100)	0/1(0)	0/1(0)	1(0.8)	NS	NS	NA
*Micronycteris* sp.	1/1(100)	0/1(0)	0/1(0)	1(0.8)	NS	NS	NA
Phyllostomidae: Phyllostominae							
*Phyllostomus discolor*	0/4(0)	4/4(100)	0/4(0)	4(3.4)	1/4(25; 0.6–80.5)	NS	NA
*Phyllostomus hastatus*	0/1(0)	0/1(0)	1/1(100)	1(0.8)	NS	NS	NA
Phyllostomidae: Stenodermatinae							
*Artibeus fimbriatus*	3/7 (42.9)	0/7(0)	4/7(57.1)	7(5.9)	1/7(14.3; 0.3–57.8)	1/7(14.3; 0.3–57.8)	1/7(14.3; 0.3–57.8)
*Artibeus lituratus*	4/14(28.6)	2/14(14.3)	8/14(57.1)	14(11.8)	1/14(7.1; 0.1–33.8)	3/14(21.4; 4.6–50.7)	NA
*Artibeus obscurus*	1/2(50)	0/2(0)	1/2(50)	2(1.7)	1/2(50; 1.2–98.7)	NS	NA
*Artibeus planirostris*	0/5(0)	5/5(100)	0/5(0)	5(4.2)	NS	NS	NA
*Chiroderma doriae*	0/1(0)	0/1(0)	1/1(100)	1(0.8)	NS	NS	NA
*Dermanura cinerea*	0/4(0)	4/4(100)	0/4(0)	4(3.4)	NS	NS	NA
*Sturnira lillium*	6/12 (50)	0/12(0)	6/12(50)	12(10.1)	6/12(50; 21.0–78.9)	NS	NA
*Sturnira tildae*	2/2(100)	0/2(0)	0/2(0)	2(1.7)	NS	NS	NA
*Vampyressa pusilla*	2/2(100)	0/2(0)	0/2(0)	2(1.7)	NS	NS	NA
Vespertilionidae: Myotinae							
*Myotis nigricans*	2/2(100)	0/2(0)	0/2(0)	2(1.7)	NS	NS	NA
TOTAL	44	47	28	119	22 (18.5; 11.9–26.6)	4 (3.4; 0.9–8.3)	1(0.8; 0.02–4.5)

*NS* negative sample, *NA* not applicable

### Detection of *Bartonella* spp.

*Bartonella* DNA was detected in 22 animals (18.5%, 95% CI: 11.9–26.6) (Table 3) collected from the three regions of this study (Table 2): Jacarepaguá/RJ (10/44; 23.0%, 95% CI: 11.4–37.8); APA Pratigi/BA (7/47; 15%, 95% CI: 6.2–28.3) and PEST/SC (5/28; 18%, 95% CI: 6.0–36.8). Although *S. lilium* (6/22 *Bartonella*-positive bats) and *D. rotundus* (6/22) were the most frequent hosts of *Bartonella*, six other bats species (i.e., *C. perspicillata, A. lituratus, A. fimbriatus, A. obscurus, R. pumilio*and *P. discolor*) were also positive (Table 3). Contrasting the pool of males against the pool of females per locality, in all study areas we found more positive samples for males than females, but only in APA Pratigi/BA this difference was significant (Fisher exact’s test *p* = 0.0027). Due to the small sample size per species/locality, we did not run this analysis per species.

**Table 3:** Specimens infected by *Bartonella* spp. and *Coxiella burnetii*. Specimens are arranged by species, locality and sex

Field Number	Species	Sex	Locality
*Bartonella* spp.			
RM 510	*Desmodus rotundus*	Female	Jacarepaguá – RJ
RM 512	*Desmodus rotundus*	Male	Jacarepaguá – RJ
RM 517	*Desmodus rotundus*	Female	Jacarepaguá – RJ
RM 523	*Desmodus rotundus*	Male	Jacarepaguá – RJ
RM 524	*Artibeus fimbriatus*	Male	Jacarepaguá - RJ
RM 525	*Sturnira lilium*	Female	Jacarepaguá - RJ
RM 529	*Artibeus obscurus*	Male	Jacarepaguá - RJ
RM 532	*Sturnira lilium*	Male	Jacarepaguá - RJ
RM 534	*Desmodus rotundus*	Male	Jacarepaguá - RJ
RM 564	*Desmodus rotundus*	Female	Jacarepaguá – RJ
EM 179	*Carollia perspicillata*	Male	APA do Pratigi - BA
EM 185	*Carollia perspicillata*	Male	APA do Pratigi - BA
EM 186	*Artibeus lituratus*	Male	APA do Pratigi - BA
EM 189	*Rhinophylla pumilio*	Male	APA do Pratigi - BA
EM 199	*Carollia perspicillata*	Male	APA do Pratigi - BA
EM 209	*Phyllostomus discolor*	Male	APA do Pratigi - BA
EM 217	*Carollia perspicillata*	Male	APA do Pratigi – BA
EM 795	*Sturnira lilium*	Male	PEST - SC
EM 800	*Carollia perspicillata*	Male	PEST - SC
EM 803	*Sturnira lilium*	Male	PEST - SC
EM 805	*Sturnira lilium*	Male	PEST - SC
EM 819	*Sturnira lilium*	Female	PEST – SC
*Coxiella burnetii*			
RM 514	*Artibeus lituratus*	Female	Jacarepaguá/RJ
RM 524	*Artibeus fimbriatus*	Male	Jacarepaguá/RJ
RM 557	*Artibeus lituratus*	Male	Jacarepaguá/RJ
EM 817	*Artibeus lituratus*	Female	PEST /SC

**Fig. 2:**
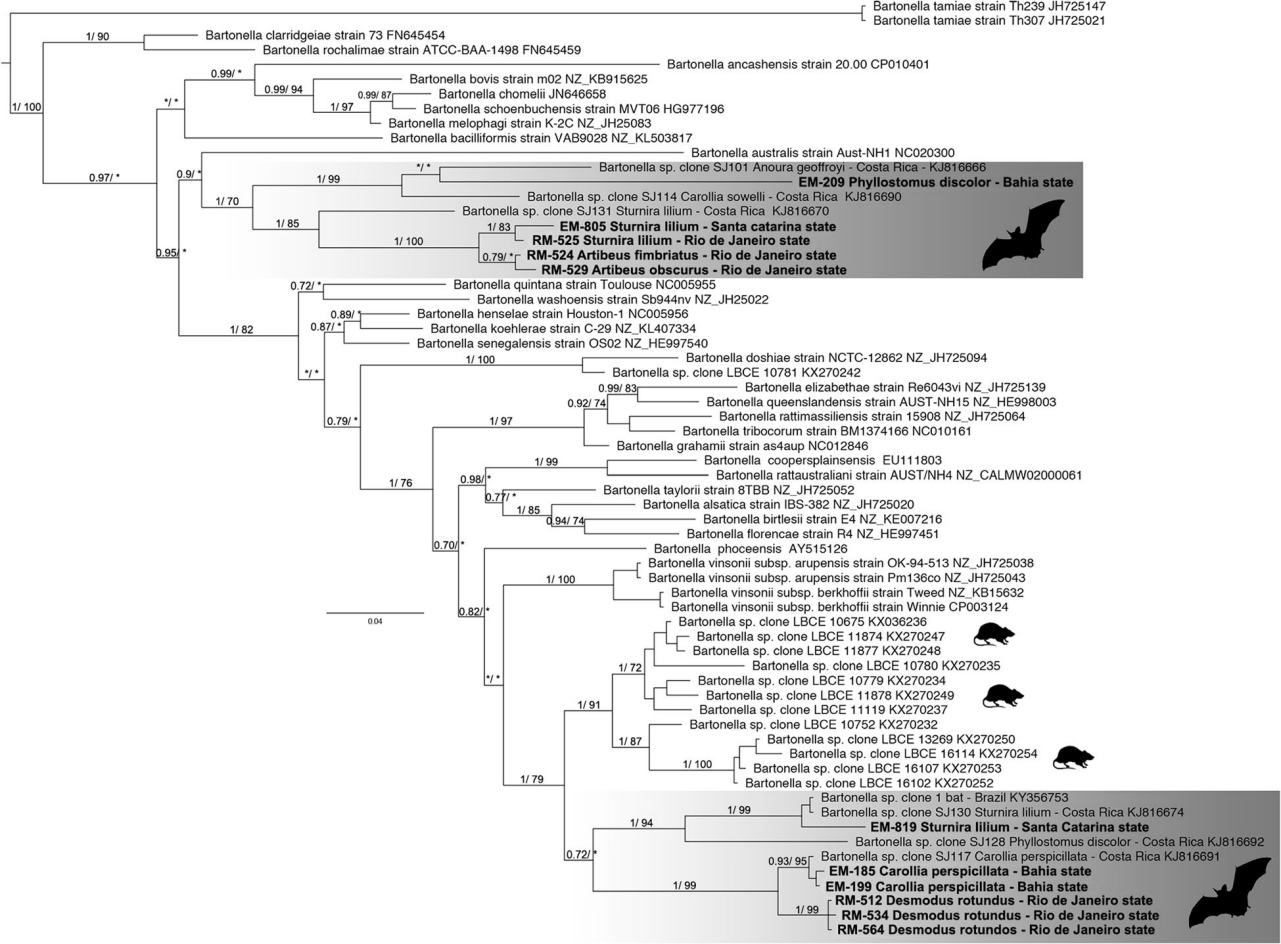
Phylogenetic relationships based on the *gltA* gene partial (512 nt) sequences of *Bartonella* species. Numbers (≥ 0.7/70%) above branches indicate posterior node probabilities or bootstrap values (Bayesian/ML). *Indicate values below 0.7/70. The Tamura three-parameter model with gamma distributed rate heterogeneity (T92 + G) was selected as the best-fit evolutionary model according to the Bayesian information criterion calculated using MEGA7 [39]. The branch labels include the GenBank accession number and the species or strain

However, in this study, we opted to work with sequences that presented fragment sizes that would allow reliable results when submitted to phylogenetic analyses. Thus, of the 22 samples that were positive for the *Bartonella gltA* gene, 11 were included in these analyses. The phylogenetic inference based on the *gltA* gene sequences revealed two different clusters for the new sequences of this study (Fig. 2). In the *gltA* gene tree, the first cluster is composed of two groups; one of them comprised our sequence (EM 209) found in *P. discolor* in the state of Bahia as well as the sequence *Bartonella* sp. clone SJ114 (KJ816690) found in *Carollia sowelli* and the sequence *Bartonella* sp. clone SJ101 (KJ816666) found in *Anoura geoffroyi*, both from Costa Rica (1/ 99). The second group in the same cluster is composed of the sequence of *Bartonella* sp. clone SJ131 (KJ816670) found in *Sturnira lilium* from Costa Rica as a stem lineage in the clade (1/ 85).The next node (1/ 100) within the cluster contained our sequences (EM 805, RM 525) found in *S. lilium* from the states of Santa Catarina and Rio de Janeiro (1/ 83), in a sister relation to the clade of our sequences found in *A. fimbriatus* and *A. obscurus* (RM 524, RM 529) from Rio de Janeiro (0.79/ *).

The second weakly supported cluster comprises two sister groups. One (1/ 99) includes our three sequences (RM 512, RM 534, RM 564) found in *D. rotundus* from the state of Rio de Janeiro, and the other includes the sequence *Bartonella* sp. clone SJ117 (KJ816691) found in *C. perspicillata* from Costa Rica and our two sequences (EM 185, EM 199) found in *C.perspicillata* from Bahia state (0.93/ 95). The other group is composed of the sequence of *Bartonella* sp. clone SJ128 (KJ816692) found in *P. discolor* from Costa Rica as a stem lineage in the group (1/ 94). The next bifurcation within the cluster contained our sequence (EM 819) found in *S. lilium* from Santa Catarina state as well as the sequence of *Bartonella* sp. clone 1 (KY356753) found in an undescribed bat species from Brazil and the sequence *Bartonella* sp. clone SJ130 (KJ816674) found *S. lilium* from Costa Rica (1/ 99).

### Detection of *Coxiella burnetii*

*Coxiella burnetii* DNA was detected in four specimens from two bat species (3.4%, 95% CI: 0.9–8.3), *A. lituratus* (3/4 Coxiella-positive bats) and *A. fimbriatus* (1/4) from two different regions: Jacarepaguá/RJ (3/44; 7%, 95% CI: 1.4–18.6) and PEST/SC (1/28; 4%, 95% CI: 0.1–18.3) (Tables 2 and 3). No differences between positivity in males and females were observed. *Coxiella* DNA sequences showed 100% identity to the complete genome of *C. burnetii* [GenBank CP018005, CP020616, AE 016828, LK 937696]. In our survey, co-infection was detected in one bat sample of *A. fimbriatus* from the Jacarepaguá/RJ region (Table 2). *Rickettsia* spp., *Ehrlichia* spp. and *Anaplasma* spp. DNA was not detected in any of the bat samples tested.

### Nucleotide sequence accession numbers

All sequences obtained including 11 for *Bartonella* spp. *gltA* (MH204887-MH204897) and 4 for *Coxiella burnetii* IS1111 (MH229948-MH229951) have been deposited in GenBank.

## Discussion

Considering the growing importance of bats as potential hosts of zoonotic agents of human disease, our findings reveal that bats of different species are infected with *Bartonella* spp. and *C. burnetii* in the Atlantic Forest regions of Rio de Janeiro, Bahia and Santa Catarina. In this study, we found a prevalence of 3.4% of bats positive for *C. burnetii* DNA, all belonging to the genus *Artibeus*. Characteristics of this genus, such as the formation of colonies grouping dozens of individuals of reproductive age [43] can contribute to a rapid and widespread transmission of *C. burnetii* among these animals, especially considering the high resistance of these proteobacteria, which can survive for several weeks in the environment where the animals were present [22]. Our results as well as recently published findings [35, 44] suggest the existence of a complex *C. burnetii* transmission cycle involving a large number of wild and domestic animals in Rio de Janeiro. In addition, the presence of *C. burnetii* DNA in bats captured in the region of Santa Catarina state is the first evidence of the circulation of this agent in the state.

Our prevalence results (18.5%) corroborate other studies worldwide showing the prevalence of *Bartonella* spp. in bats between 18.0–33.3% [10, 24, 27]. In our study, *S. lilium* (27.3%), *D. rotundus* (27.3%) and *C. perspicillata* (22.7%) were the species that presented the highest prevalence of *Bartonella* infection. In South America, similar results were obtained in Peru, with a prevalence of 15% for *C. perspicillata* (4/27), 37% for *D. rotundus*, (10/27) and 4% for *S. lilium* (1/27) [24]. In Brazil, in a study recently carried out in 5 different states, positive samples for *Bartonella* spp. were found in *S. lilium*, *C. perspicillata*, *P. discolor*, *Glossophaga soricina* and *Natalus espiritosantensis* (*Natalusmacrourus* [45]) [11]. Although we have found more positive samples for males than females in all studied areas, the evidence available, including the established knowledge on transmission routes and the role of bats in the circulation of these pathogens, do not allow us to speculate on the pathogen prevalence in males. Further analyses comparing males and females per species are necessary for better understanding the role of sexes in the pathogen circulation.

The phylogenetic tree revealed the formation of independent clades when compared to *Bartonella* species reported in the literature, which may indicate that previously unknown genotypes of *Bartonella* are infecting these bats. This is a common finding with studies of this agent in bats from other regions of the world (e.g., in Guatemala, Nigeria, Costa Rica and China) [7, 10, 27, 46]. Some of our obtained sequences showed a clear separation of the group formed in *Bartonella* sequences found in wild rodents of the Atlantic Forest in the state of Rio de Janeiro (LBCE- Laboratory of Biology and Control of Schistosomiasis), reinforcing that a new genotype may be circulating among bats and that these strains differ between rodents and bats [35]. Interestingly, our sequences were most closely related to others identified in bats from Costa Rica and related only to a single sequence found in a Brazilian bat. Furthermore, positive samples belonging to the *S. lilium* species were grouped into two distinct clades, suggesting that a single bat species is host to two different species of *Bartonella*. Similarly, sequences generated from Bahia, Rio de Janeiro and Santa Catarina states subjected to phylogenetic analyses were divided into two clades and suggested that the circulation of more than one species of *Bartonella* may be associated with bats in each state. Co-infection of different *Bartonella* species in a single bat species was also observed in Kenya, Guatemala, China and Georgia [25, 27, 46, 47]. A recent study demonstrated that *Bartonella* strains tend to cluster according to families, super-families and suborders of bats [48]. In addition, a co-infection with different species of *Bartonella* in a single species of bat may imply a change of bacteria via recombination, as shown by *Bartonella* in rodents [49]. The presence of divergent sequences in the analyzes of this study suggests the presence of more than one *Bartonella* lineage, since the divergence of the sampled sequences of the *gltA* gene varied from 0.0 to 20.9% in sequences of the same clade, the sequence divergence among Bartonella species suggested for this fragment is about 30% [50] (Additional file 1). To characterize the *Bartonella* species, it is necessary to sequencing other housekeeping genes (ie, *rpoB*, *ftsZ*,*groEL* and its) to recognize the diversity of lineages found in bats and to determine the structure of populations and phylogenetic data [51, 52]. Sequencing of the additional genes for improved taxonomic resolution was beyond the scope of this paper.

Our study has identified a co-infection with *C. burnetii* and *Bartonella* spp. in an individual of *A. fimbriatus*. Although a pattern cannot be established, dual infection can reinforce the potential of bats to host these bacterial pathogens. Many bat species are gregarious and can form small groups of a few individuals to large colonies of up to 20 million individuals, such as the species *Tadarida brasiliensis* in Bracken Cave in Texas, USA [53]. Furthermore, different bat species can cohabitate in the same shelter, allowing the possibility for interspecific transmission and a high rate of contact within these colonies that can lead to rapid transmission of pathogens [54]. Bats have been identified as potential natural reservoirs of a number of high-impact zoonotic agents. Recently, a study provided evidence that bats are indeed special in hosting more zoonotic viruses and more total viruses per species than rodents [55].

The absence of *Rickettsia* in bat samples corroborate the literature demonstrating that a lack of rickettsial amplification in wild animal is an expected result, since vertebrates act as amplifiers and food sources for ticks, which are in fact the true reservoirs of these proteobacteria in nature [35]. Besides, this reinforces that the role of bats as carriers of *Rickettsia* is still unknown, despite reports in the 1950s that bats harbor pathogenic rickettsial [19, 56, 57]. No samples tested in our study were positive for *Ehrlichia* and *Anaplasma* infections. Although bats have been found infected with proteobacteria of the families Anaplasmataceae [58], there is still no record of DNA amplification of *Ehrlichia* spp. or *Anaplasma* spp. in these mammals. Regardless of which other prior studies have used a similar method, it seems plausible that some negative detections may have resulted from a less sensitive PCR approach (i.e., conventional rather than nested).

## Conclusion

This study confirms the presence of infected bats with *C. burnetii* and *Bartonella* spp. in the Brazilian Atlantic Forest. To the best of our knowledge, this is the first study that reports *C. burnetii* infection in Brazilian bats and the first to report *Bartonella* spp. in the Atlantic Forest biome.

## Additional file

Additional file 1: Estimates of Evolutionary Divergence between *Bartonella gltA* partial sequences. There were a total of 512 positions in the final dataset. Evolutionary analyses were conducted in MEGA7. Presentation of the PCR positive bats species for *Bartonella* spp. in this study with their respective GenBank accession numbers and the estimated divergence found between *Bartonella gltA* partial sequences of the gene deposited in GenBank. (DOCX 25 kb)

## Abbreviations

APA: Environmental Protection Area
BA: Bahia
CEUA: Ethics Committee on Animal Research
CIs: Confidence intervals
CNPq: Conselho Nacional para o Desenvolvimento Cientifico e Tecnológico
EFMA: Fiocruz Atlantic Forest Biological Station
FAPERJ: Fundação de Amparo à Pesquisa do Estado do Rio de Janeiro
FATMA: Santa Catarina’s Environment Foundation
Fiocruz: Oswaldo Cruz Foundation
IBAMA: Brazilian Institute of Environment and Renewable Natural Resources
ICMBio/SISBIO: Chico Mendes Biodiversity Conservation Institute
LBCE: Laboratory of Biology and Control of Schistosomiasis
MCMC: Bayesian Markov Chain Monte Carlo
PAPES: Strategic Health Research Program
PCR: Polymerase chain reaction
PEST: Serra do Tabuleiro State Park
PPBIO: Biodiversity Research Program
RJ: Rio de Janeiro
SC: Santa Catarina
