# Supplementary material for: Coxiella and Bartonella spp. in bats (Chiroptera) captured in the Brazilian Atlantic Forest biome
Source: BMC Vet Res. 2018 Sep 10;14:279. doi: 10.1186/s12917-018-1603-0 (PMC6131887; doi:10.1186/s12917-018-1603-0)
Supplement: Supplementary file 1 — Estimates of Evolutionary Divergence between Bartonella gltA partial sequences. There were a total of 512 positions in the final dataset. Evolutionary analyses were conducted in MEGA7. Presentation of the PCR positive bats species for Bartonella spp. in this study with their respective GenBank accession numbers and the estimated divergence found between Bartonella gltA partial sequences of the gene deposited in GenBank. (DOCX 25 kb) [file 12917_2018_1603_MOESM1_ESM.docx]

Additional file 01. Estimates of Evolutionary Divergence between *Bartonella* *gltA* partial sequences. There were a total of 512 positions in the final dataset. Evolutionary analyses were conducted in MEGA7[39].

| **Species 1 (Genbank no.)** | **Species 2 (Genbank no.)** | **p Distance** |
| --- | --- | --- |
| **CLADE 1** | | |
| EM 209 Phyllostomus discolor Bahia (MH204889) | Bartonella australis strain Aust NH1 (NC020300) | 20.9% |
| EM 209 Phyllostomus discolor Bahia (MH204889) | Bartonella sp. clone SJ101 (KJ816666) | 13.7% |
| EM 209 Phyllostomus discolor Bahia (MH204889) | Bartonella sp. clone SJ114 (KJ816690) | 12.7% |
| EM 209 Phyllostomus discolor Bahia (MH204889) | Bartonella sp. clone SJ131 (KJ816670) | 14.6% |
| EM 805 Sturnira lilium Santa Catarina (MH204890) | Bartonella australis strain Aust NH1 (NC020300) | 18.2% |
| EM 805 Sturnira lilium Santa Catarina (MH204890) | Bartonella sp. clone SJ101 (KJ816666) | 14.1% |
| EM 805 Sturnira lilium Santa Catarina (MH204890) | Bartonella sp. clone SJ114 (KJ816690) | 15.4% |
| EM 805 Sturnira lilium Santa Catarina (MH204890) | Bartonella sp. clone SJ131 (KJ816670) | 10.4% |
| EM 805 Sturnira lilium Santa Catarina (MH204890) | EM 209 Phyllostomus discolor Bahia (MH204889) | 17.2% |
| RM 524 Artibeus fimbriatus Rio de Janeiro (MH204895) | Bartonella australis strain Aust NH1 (NC020300) | 17.8% |
| RM 524 Artibeus fimbriatus Rio de Janeiro (MH204895) | Bartonella sp. clone SJ101 (KJ816666) | 13.3% |
| RM 524 Artibeus fimbriatus Rio de Janeiro (MH204895) | Bartonella sp. clone SJ114 (KJ816690) | 13.9% |
| RM 524 Artibeus fimbriatus Rio de Janeiro (MH204895) | Bartonella sp. clone SJ131 (KJ816670) | 10.0% |
| RM 524 Artibeus fimbriatus Rio de Janeiro (MH204895) | EM 209 Phyllostomus discolor Bahia (MH204889) | 16.2% |
| RM 524 Artibeus fimbriatus Rio de Janeiro (MH204895) | EM 805 Sturnira lilium Santa Catarina (MH204890) | 3.7% |
| RM 525 Sturnira lilium Rio de Janeiro (MH204896) | Bartonella australis strain Aust NH1 (NC020300) | 18.2% |
| RM 525 Sturnira lilium Rio de Janeiro (MH204896) | Bartonella sp. clone SJ101 (KJ816666) | 13.7% |
| RM 525 Sturnira lilium Rio de Janeiro (MH204896) | Bartonella sp. clone SJ114 (KJ816690) | 14.8% |
| RM 525 Sturnira lilium Rio de Janeiro (MH204896) | Bartonella sp. clone SJ131 (KJ816670) | 9.6% |
| RM 525 Sturnira lilium Rio de Janeiro (MH204896) | EM 209 Phyllostomus discolor Bahia (MH204889) | 16.4% |
| RM 525 Sturnira lilium Rio de Janeiro (MH204896) | EM 805 Sturnira lilium Santa Catarina (MH204890) | 1.6% |
| RM 525 Sturnira lilium Rio de Janeiro (MH204896) | RM 524 Artibeus fimbriatus Rio de Janeiro (MH204895) | 2.5% |
| RM 529 Artibeus obscurus Rio de Janeiro (MH204897) | Bartonella australis strain Aust NH1 (NC020300) | 18.2% |
| RM 529 Artibeus obscurus Rio de Janeiro (MH204897) | Bartonella sp. clone SJ101 (KJ816666) | 13.5% |
| RM 529 Artibeus obscurus Rio de Janeiro (MH204897) | Bartonella sp. clone SJ114 (KJ816690) | 14.1% |
| RM 529 Artibeus obscurus Rio de Janeiro (MH204897) | Bartonella sp. clone SJ131 (KJ816670) | 10.2% |
| RM 529 Artibeus obscurus Rio de Janeiro (MH204897) | EM 209 Phyllostomus discolor Bahia (MH204889) | 16.6% |
| RM 529 Artibeus obscurus Rio de Janeiro (MH204897) | EM 805 Sturnira lilium Santa Catarina (MH204890) | 4.3% |
| RM 529 Artibeus obscurus Rio de Janeiro (MH204897) | RM 524 Artibeus fimbriatus Rio de Janeiro (MH204895) | 0.8% |
| RM 529 Artibeus obscurus Rio de Janeiro (MH204897) | RM 525 Sturnira lilium Rio de Janeiro (MH204896) | 3.1% |
| Bartonella sp. clone SJ101 (KJ816666) | Bartonella australis strain Aust NH1 (NC020300) | 18.9% |
| Bartonella sp. clone SJ114 (KJ816690) | Bartonella australis strain Aust NH1 (NC020300) | 18.8% |
| Bartonella sp. clone SJ114 (KJ816690) | Bartonella sp. clone SJ101 (KJ816666) | 8.8% |
| Bartonella sp. clone SJ131 (KJ816670) | Bartonella australis strain Aust NH1 (NC020300) | 18.4% |
| Bartonella sp. clone SJ131 (KJ816670) | Bartonella sp. clone SJ101 (KJ816666) | 13.1% |
| Bartonella sp. clone SJ131 (KJ816670) | Bartonella sp. clone SJ114 (KJ816690) | 13.3% |
| **CLADE 2** | | |
| EM 185 Carollia perspicillata Bahia (MH204887) | Bartonella sp. clone 1 (KY356753) | 10.9% |
| EM 185 Carollia perspicillata Bahia (MH204887) | Bartonella sp. clone SJ117 (KJ816691) | 0.4% |
| EM 185 Carollia perspicillata Bahia (MH204887) | Bartonella sp. clone SJ128 (KJ816692) | 11.5% |
| EM 185 Carollia perspicillata Bahia (MH204887) | Bartonella sp. clone SJ130 (KJ816674) | 10.9% |
| EM 199 Carollia perspicillata Bahia (MH204888) | Bartonella sp. clone 1 (KY356753) | 10.7% |
| EM 199 Carollia perspicillata Bahia (MH204888) | Bartonella sp. clone SJ117 (KJ816691) | 0.2% |
| EM 199 Carollia perspicillata Bahia (MH204888) | Bartonella sp. clone SJ128 (KJ816692) | 11.3% |
| EM 199 Carollia perspicillata Bahia (MH204888) | Bartonella sp. clone SJ130 (KJ816674) | 10.7% |
| EM 199 Carollia perspicillata Bahia (MH204888) | EM 185 Carollia perspicillata Bahia (MH204887) | 0.2% |
| EM 819 Sturnira lilium Santa Catarina (MH204894) | Bartonella sp. clone 1 (KY356753) | 2.5% |
| EM 819 Sturnira lilium Santa Catarina (MH204894) | Bartonella sp. clone SJ117 (KJ816691) | 11.9% |
| EM 819 Sturnira lilium Santa Catarina (MH204894) | Bartonella sp. clone SJ128 (KJ816692) | 10.4% |
| EM 819 Sturnira lilium Santa Catarina (MH204894) | Bartonella sp. clone SJ130 (KJ816674) | 2.5% |
| EM 819 Sturnira lilium Santa Catarina (MH204894) | EM 185 Carollia perspicillata Bahia (MH204887) | 12.3% |
| EM 819 Sturnira lilium Santa Catarina (MH204894) | EM 199 Carollia perspicillata Bahia (MH204888) | 12.1% |
| RM 512 Desmodus rotundus Rio de Janeiro (MH204891) | Bartonella sp. clone 1 (KY356753) | 11.9% |
| RM 512 Desmodus rotundus Rio de Janeiro (MH204891) | Bartonella sp. clone SJ117 (KJ816691) | 2.9% |
| RM 512 Desmodus rotundus Rio de Janeiro (MH204891) | Bartonella sp. clone SJ128 (KJ816692) | 12.9% |
| RM 512 Desmodus rotundus Rio de Janeiro (MH204891) | Bartonella sp. clone SJ130 (KJ816674) | 11.9% |
| RM 512 Desmodus rotundus Rio de Janeiro (MH204891) | EM 185 Carollia perspicillata Bahia (MH204887) | 3.3% |
| RM 512 Desmodus rotundus Rio de Janeiro (MH204891) | EM 199 Carollia perspicillata Bahia (MH204888) | 3.1% |
| RM 512 Desmodus rotundus Rio de Janeiro (MH204891) | EM 819 Sturnira lilium Santa Catarina (MH204894) | 12.5% |
| RM 534 Desmodus rotundus Rio de Janeiro (MH204892) | Bartonella sp. clone 1 (KY356753) | 12.1% |
| RM 534 Desmodus rotundus Rio de Janeiro (MH204892) | Bartonella sp. clone SJ117 (KJ816691) | 3.1% |
| RM 534 Desmodus rotundus Rio de Janeiro (MH204892) | Bartonella sp. clone SJ128 (KJ816692) | 13.1% |
| RM 534 Desmodus rotundus Rio de Janeiro (MH204892) | Bartonella sp. clone SJ130 (KJ816674) | 12.1% |
| RM 534 Desmodus rotundus Rio de Janeiro (MH204892) | EM 185 Carollia perspicillata Bahia (MH204887) | 3.5% |
| RM 534 Desmodus rotundus Rio de Janeiro (MH204892) | EM 199 Carollia perspicillata Bahia (MH204888) | 3.3% |
| RM 534 Desmodus rotundus Rio de Janeiro (MH204892) | EM 819 Sturnira lilium Santa Catarina (MH204894) | 12.3% |
| RM 534 Desmodus rotundus Rio de Janeiro (MH204892) | RM 512 Desmodus rotundus Rio de Janeiro (MH204891) | 0.2% |
| RM 564 Desmodus rotundos Rio de Janeiro (MH204893) | Bartonella sp. clone 1 (KY356753) | 11.9% |
| RM 564 Desmodus rotundos Rio de Janeiro (MH204893) | Bartonella sp. clone SJ117 (KJ816691) | 2.9% |
| RM 564 Desmodus rotundos Rio de Janeiro (MH204893) | Bartonella sp. clone SJ128 (KJ816692) | 12.9% |
| RM 564 Desmodus rotundos Rio de Janeiro (MH204893) | Bartonella sp. clone SJ130 (KJ816674) | 11.9% |
| RM 564 Desmodus rotundos Rio de Janeiro (MH204893) | EM 185 Carollia perspicillata Bahia (MH204887) | 3.3% |
| RM 564 Desmodus rotundos Rio de Janeiro (MH204893) | EM 199 Carollia perspicillata Bahia (MH204888) | 3.1% |
| RM 564 Desmodus rotundos Rio de Janeiro (MH204893) | EM 819 Sturnira lilium Santa Catarina (MH204894) | 12.5% |
| RM 564 Desmodus rotundos Rio de Janeiro (MH204893) | RM 512 Desmodus rotundus Rio de Janeiro (MH204891) | 0.0% |
| RM 564 Desmodus rotundos Rio de Janeiro (MH204893) | RM 534 Desmodus rotundus Rio de Janeiro (MH204892) | 0.2% |
| Bartonella sp. clone SJ117 (KJ816691) | Bartonella sp. clone 1 (KY356753) | 10.5% |
| Bartonella sp. clone SJ128 (KJ816692) | Bartonella sp. clone 1 (KY356753) | 8.6% |
| Bartonella sp. clone SJ128 (KJ816692) | Bartonella sp. clone SJ117 (KJ816691) | 11.1% |
| Bartonella sp. clone SJ130 (KJ816674) | Bartonella sp. clone 1 (KY356753) | 0.0% |
| Bartonella sp. clone SJ130 (KJ816674) | Bartonella sp. clone SJ117 (KJ816691) | 10.5% |
| Bartonella sp. clone SJ130 (KJ816674) | Bartonella sp. clone SJ128 (KJ816692) | 8.6% |
